# Multitrait indices to predict worm length and number in sheep with natural, mixed predominantly *Teladorsagia circumcincta* infection

**DOI:** 10.1017/S0031182014001905

**Published:** 2015-01-14

**Authors:** COLETTE MAIR, LOUISE MATTHEWS, JOAQUIN PRADA J. DE CISNEROS, THORSTEN STEFAN, MICHAEL J. STEAR

**Affiliations:** Institute of Biodiversity, Animal Health & Comparative Medicine, University of Glasgow, Bearsden Road, Glasgow G61 1QH, UK

**Keywords:** nematode infection, worm number, worm length, predictive index

## Abstract

Accurately identifying resistance to gastrointestinal nematode infections requires the ability to identify animals with low and high intensities of infection. The pathogenic effects of nematodes depend upon both the length and number of worms, neither of which can be measured in live animals. Indices that predict these quantities are urgently needed. Monthly fecal egg counts, bodyweights, IgA concentrations and pepsinogen concentrations were measured on Scottish Blackface sheep naturally infected with a mixture of nematodes, predominantly *Teladorsagia circumcincta*. Worm number and average worm length were available on over 500 necropsied lambs. We derived predictive indices for worm length and number using linear combinations of traits measured in live animals. The correlations between the prediction values and the observed values were 0·55 for worm length and 0·51 for worm number. These indices can be used to identify the most resistance and susceptible lambs.

## INTRODUCTION

Nematodes reduce animal health and welfare as well as the profitability of livestock production. In predominantly *Teladorsagia circumcincta* infection, the major consequences are reduced growth and occasionally the death of severely affected animals. Profitable production of small ruminants requires the control of nematodes, for example through selective breeding or targeted selective treatment. Effective implementation of such controls requires a better understanding of the host–parasite interaction, and one of the major constraints is our inability to determine the intensity of infection in a live animal.

The severity of infection depends on the nutritional state of the host and its ability to mount an immune response (Stear *et al*. 2003; Roeber *et al*. 2013). Resistance is defined as the ability of a host to suppress establishment and/or development of a parasite (Sayers and Sweeney, 2005). Thus, resistance can be assessed directly by measuring worm number and worm length (Stear *et al.* 1999*b*; Sayers and Sweeney, 2005). Neither can be measured in live animals.

Worm number is a direct method of identifying resistant animals (Sayers and Sweeney, 2005) and is correlated with an animal's productivity. Consequently, inaccurate estimation of worm number can hamper disease management (Raadsma *et al*. 2008). In contrast, worm length can be measured more accurately (Stear *et al.* 1999*a*) and is a more heritable trait with resistant lambs able to better control worm growth rather than worm numbers (Strain *et al*. 2002).

Several traits have been shown to correlate with nematode infection (Davies *et al*. 2005), one of which is fecal egg count (FEC), which is routinely used as a measure of resistance (Sayers and Sweeney, 2005). The number of eggs reflects both the number of worms present and their fecundity, which depends on worm length (Stear *et al.* 2003). Several other traits are also associated with *T. circumcincta* worm number and length. Increased concentrations of parasite-specific mucosal IgA and eosinophil are associated with reduced adult worm length, suggesting that worm length is regulated through an interaction between IgA and eosinophils (Henderson and Stear, 2006). Plasma pepsinogen concentration (Peps) is associated with abomasal damage as well as the immune response. It is associated with worm length and weakly associated with worm number (Stear *et al*. 1999*a*). Reduced weight gain is also a characteristic of infected lambs (Miller and Horohov, 2006) and is used as a measure of animal resilience (Bisset and Morris, 1996). As a result, the degree of infection manifests itself through a complex interaction between many traits and a multitrait marker may be a valuable tool for identifying resistant sheep. The aim of the present paper is to describe the creation of predictive indices for worm number and worm length by combining several immunological and parasitological traits that are associated with nematode infection.

## MATERIALS AND METHODS

### Data

Data were collected over consecutive years from five cohorts of 200 straight-bred Scottish Blackface lambs. All lambs were given anthelmintic every 28 days. Within each year, monthly FEC, weights, IgA concentrations, Peps and eosinophilia were measured using standard procedures between May and October with additional post-mortem counts (Stear *et al*. 1999*a*; Strain *et al*. 2002; Henderson and Stear, 2006). In total, 20 different measurements were collected on live animals. However, the data are unbalanced with different numbers of animals tested for each trait (Table 1). Summary statistics for each trait in the month of October are provided by Davies *et al*. (2005).

**Table 1.** Summary statistics for 20 predictor variables including median values, ranges (minimal and maximal values) and the per cent of missing values from the 490 necropsied lambs

Trait	Median	Range	Per cent missing (%)	Trait	Median	Range	Per cent missing (%)
May fecal egg count (FEC.1)	0	(0, 8100)	61	May weight (WT1)	10	(4, 17·5)	2
June fecal egg count (FEC.2)	200	(0, 1750)	42	June weight (WT2)	17	(9, 25)	2
July fecal egg count (FEC.3)	350	(0, 3200)	13	July weight (WT3)	22	(12, 36)	0·6
Aug fecal egg count (FEC.4)	150	(0, 2450)	9	Aug weight (WT4)	27	(14, 38)	2
Sep fecal egg count (FEC.5)	125	(0, 2700)	4	Sep weight (WT5)	29	(16, 43)	1
Oct fecal egg count (FEC.6)	225	(0, 3612)	27	May eosinophil (EOS1)	3	(0, 56)	80
August IgA (IgA.4)	0·06	(0, 1·38)	31	June eosinophil (EOS2)	3	(0, 59)	80
Sep IgA (IgA.5)	0·15	(0, 0·87)	0·8	July eosinophil (EOS3)	3	(0, 40)	80
Oct IgA (IgA.6)	0·1	(0, 0·79)	44	Aug eosinophil (EOS4)	9	(0, 83)	60
Oct pepsinogen (Peps)	21·6	(0, 281·4)	3	Sep eosinophil (EOS5)	9	(0, 161)	50

Post-mortem adult worm number and length were collected from 531 lambs. After data editing to remove lambs with incomplete information 490 lambs remained. In total seven categories of adult nematode were found with variation in prevalence across the five years. *T. circumcincta* were found in all lambs (Stear *et al.* 1998). We use worm number and worm length to refer to *T. circumcincta* only. The distribution of adult *T. circumcincta* is overdispersed and follows a negative binomial distribution (Henderson and Stear, 2006). Adult worm length was defined as the average length of at least 25 randomly selected adult female worms, and had a distribution consistent with a normal distribution (Stear *et al.* 2007).

### Univariate analysis

We assessed the linear relationships between each response variable and the list of independent variables (Table 1) using a series of linear regressions. For each response, the *p*-values were corrected for multiple comparisons using the methods described by Bretz *et al*. (2011). The *p*-values were adjusted using the *p*-adjust function in R (R Core Team, 2013).

In addition to the original variables (Table 1), we also considered other possible indicators of infection. The relationship between worm number and FEC is not linear (Bishop and Stear, 2000); low FECs occur in animals with both low and high worm numbers. Therefore, quadratic relationships between FECs and worm length and number were considered. To predict worm number and worm length, we considered changes in lamb weights in July and August, and August and September. Lastly, there may be interactions between some of the variables. Increased IgA activity is negatively correlated with FECs (Strain *et al*. 2002). Interaction terms between FECs and IgA across the 6 months were included.

### Multivariate analyses

Multiple linear regression (MR) is a commonly used method for estimating the importance of multiple independent variables in accounting for variation in a dependent variable. It is also used for prediction (Raadsma *et al.* 2008; Nathans *et al*. 2012). In this paper, we take a regression approach to predict the intensity of nematode infection in lambs using a range of traits. However, many of the potential predictive traits are correlated. Some correlations arise because the data are longitudinal. Others arise for biological reasons. For example, plasma IgA and peripheral eosinophilia have similar kinetics (Henderson and Stear, 2006). High correlations between variables can cause multicollinearity problems by inflating estimated standard errors of regression coefficients (Dunteman, 1989) creating difficulty in finding statistically significant variables. Principal component analysis (PCA) is a dimension reduction tool that can be used in these circumstances. In multivariate problems, PCA aims to make independent linear combinations of the original variables which account for most of the variation in the data, and correlation principal component regression (CPCR) can be used as a multivariate calibration method to overcome multicollinearities between regression variables (Jolliffe, 2002).

For both responses – worm number and length – we performed PCA on the full set of variables, reducing the dimensionality to relatively few components. By selecting the components most correlated to the response variable and analysing each component weight, or loading, this method assesses the importance of each variable in prediction (Magidson, 2013).

Our interest lies primarily in the predictive ability of each regression model. The model selection criteria used was root mean squared error of prediction (RMSEP) for worm length and root mean squared log error of prediction (RMSLEP) for worm number (described below). For each response, we compared 6 models, three versions of the MR model and three versions of the CPCR model (Table 2). Specifically, the alternative versions allowed us to compare the predictive ability of the full set of variables (models MR3 and CPCR3) with the set of variables found to be significant in the univariate analyses (models MR2 and CPCR2) in addition to assessing the value of repeated measurements (models MR1 and CPCR1).

**Table 2.** Description of the 6 models applied to the two variables

Code	Method	Response	Variables
MR1_Length	Multiple regression	Worm length	Last measurement taken for each variable
CPCR1_Length	Correlation principal components regression	Worm length	Last measurement taken for each variable
MR2_Length	Multiple regression	Worm length	Variables found to be significant listed in Table 3
CPCR2_Length	Correlation principal components regression	Worm length	Variables found to be significant listed in Table 3
MR3_Length	Multiple regression	Worm length	Full set of variables
CPRR3_Length	Correlation principal components regression	Worm length	Full set of variables
MR1_Number	Multiple regression	Worm number	Last measurement taken for each variable
CPCR1_Number	Correlation principal components regression	Worm number	Last measurement taken for each variable
MR2_Number	Multiple regression	Worm number	Variables found to be significant listed in Table 4
CPCR2_Number	Correlation principal components regression	Worm number	Variables found to be significant listed in Table 4
MR3_Number	Multiple regression	Worm number	Full set of variables
CPRR3_Number	Correlation principal components regression	Worm number	Full set of variables

**Table 3.** Univariate significant relationships found between worm length and the set of predictor variables (corrected for sex and year of birth). For each variable, the correlation and RMSEP are given. The *p*-values reported for quadratic relationships correspond to the quadratic term

Variable	Direction	*p*-value	Correlation	RMSEP
IgA.4	−	0·027	−0·17 (−0·27, −0·07)	0·017 (0·015, 0·019)
IgA.5	−	0·0007	−0·18 (−0·27, −0·10)	0·016 (0·015, 0·018)
FEC.4	+	<0·0001	0·23 (0·14, 0·31)	0·017 (0·015, 0·018)
FEC.5	+	0·0007	0·19 (0·10, 0·28)	0·017 (0·015, 0·018)
WT.4	+	0·001	0·18 (0·09, 0·27)	0·017 (0·015, 0·019)
WT.5	+	0·036	0·14 (0·05, 0·23)	0·017 (0·015, 0·019)
Peps	−	<0·0001	−0·34 (−0·42, −0·27)	0·016 (0·014, 0·018)
FEC.4 +FEC.4^2^	+	0·02		0·017 (0·015, 0·018)
WT.4-WT.3	+	0·00053	0·19 (0·11, 0·28)	0·017 (0·015, 0·019)

**Table 4.** Univariate significant relationships found between worm number and the set of predictor variables (corrected for sex and year of birth). For each variable, the correlation and RMSLEP are given. The *p*-values reported for quadratic relationships correspond to the quadratic term

Variable	Direction	*p*-value	Correlation	RMSLEP
IgA.4	+	<0·0001	0·19 (0·08, 0·29)	1·09 (0·84, 1·30)
FEC.6	+	<0·0001	0·30 (0·20, 0·39)	0·99 (0·75, 1·18)
WT.2	−	0·019	−0·10 (−0·19, −0·01)	1·11 (0·83, 1·31)
WT.3	−	0·004	−0·13 (−0·21, −0·04	1·10 (0·83, 1·32)
WT.4	−	<0·0001	−0·24 (−0·33, −0·16)	1·09 (0·83, 1·30)
WT.5	−	<0·0001	−0·27 (−0·35, −0·19)	1·08 (0·81, 1·29)
Peps	+	<0·0001	0·37 (0·29, 0·45)	1·09 (0·82, 1·28)
FEC.6 **×** IgA.6	+	0·011	0·20 (0·08, 0·32)	1·05 (0·81, 1·27)
FEC.6 + FEC.6^2^	+	0·0005	–	0·97 (0·73, 1·14)
WT.4-WT.3	−	<0·0001	−0·23 (−0·32, −0·15)	1·08 (0·83, 1·31)

### Correlation principal component regression

The method used was adapted from Sun (1995). Given a training dataset of size *n* with response variable *Y* and *P* explanatory variables stored in matrix *X* of dimension *n* × *P*:

1. For variable *X*_*j*_ (*j*= 1…*P*), set 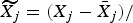 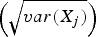. This produces a matrix 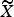, a scaled and centred version of *X*.
2. Find a set of linear combinations *PC*_1_…*PC*_*P*_ such that for lamb *i*,

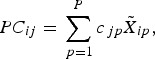

where *c*_*jp*_ are the component loadings.
3. Regress *Y* on the *A* principal components, *PC*_1_…*PC*_*A*_, most correlated with *Y*. Selecting the value of *A* is discussed in the next section.
4. Predict new observation *y*′ by

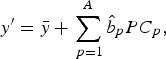

where 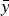 is the mean value of the response variable Y and 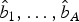 are estimated regression coefficients.

### Model selection

To find the best predictor of worm number or worm length, we used 10-fold cross-validation to select the value of *A* (step 3 in CPCR) which minimized the RMSEP, defined as
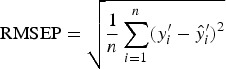


(Sun, 1995). This is a function of the difference between the true value 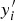 and the predicted value 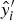 with each difference equally weighted. The RMSLEP
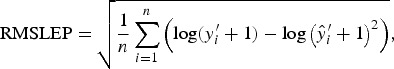
is more appropriate for data over a larger range. Worm numbers in this data are between 100 and 28 400. Therefore, any slight deviations from the true values in the upper tail of this distribution will heavily influence the value of RMSEP. We therefore used RMSEP to choose the value of *A* when modelling an average worm length and we used RMSLEP when modelling a worm number.

In order to compare the results from the 6 models (Table 2), we randomly sampled 300 observations to act as a training set and the remaining data were used as a test set and RMSEP (or RMSLEP in the case of worm number) was calculated to access the predictive ability of the models. This process was repeated 1000 times. RMSEP and RMSELP are only informative about the accuracy of a predictor.

To assess precision, we computed prediction bands. For any linear regression model, uncertainty arises due to the variability between the fitted values and the observed values. However, given that the fitted values are only expectations of the observed data, applying a fitted regression model to new data presents additional sources of variation due to the uncertainty in these expectations (Gelman *et al*. 2004). Since we assumed normality in modelling worm length, we computed prediction intervals in R using the *predict.lm* function. However, we took a Bayesian approach to compute prediction bands for worm number since we used negative binomial models. This was achieved by first sampling from the joint posterior distribution of all estimated regression parameters given in the observed data, and given these samples and new data, we simulated from the data distribution (Gelman *et al*. 2004).

The best model minimized RMSEP (or RMSLEP) and produced narrow prediction bands.

### Rotating principal components

Principal components are useful if they are interpretable and rotating a set of components can ease interpretation (Jolliffe, 2002). Variables that are most influential in prediction are more heavily weighted in the first component, which is the component most correlated with the response variable, and have previously been referred to as prime predictors (Magidson, 2013). We rotated the set of components using the varimax method, which is designed to inflate higher component weights and decrease the lower weights (Dunteman, 1989).

### Missing data

A major constraint in using principal component regression is that we require observations from all of the original variables for all of the lambs, which is not the case in the dataset (Table 1). One method is to simply use only lambs with a complete set of observations in any analysis. However, very few lambs had observations for every variable. There are several statistical methods for imputation of missing data. We used singular value decomposition (Troyanskaya *et al*. 2001 and Wong, 2013).

## RESULTS

We first analysed the data univariately and then considered the full set of variables simultaneously.

### Univariate analysis

**Fig. 1.**
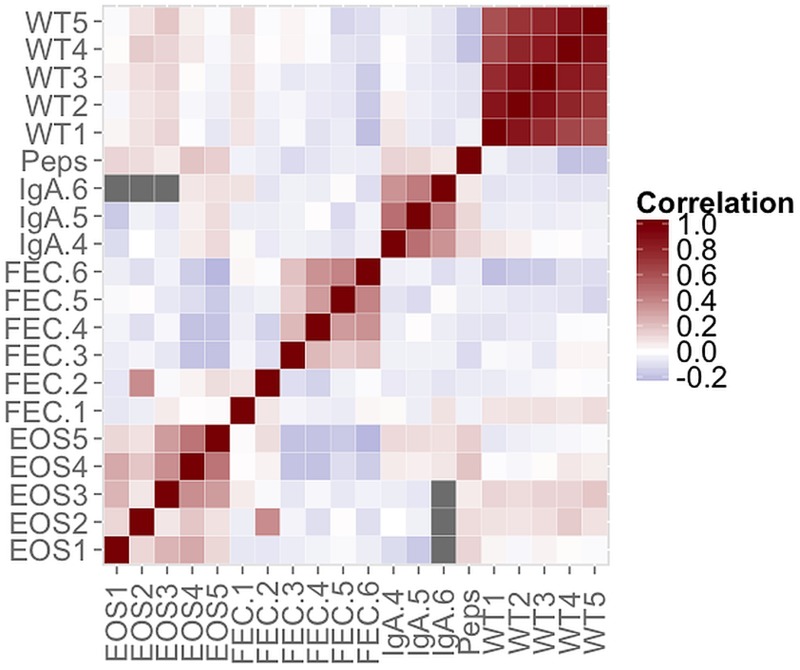
Pairwise correlations between variables listed in Table 1. Correlations between IgA.6 and EOS1, EOS2 and EOS3 could not be estimated due to the overlap in missing data (black boxes). Grey boxes indicate non-significant correlations.

Pairwise correlations between the variables are shown in Fig. 1. The highest correlations occurred between repeated measurements of the same variable. For instance, lamb weights between May and September had correlations from 0·89 between June and July to 0·62 between May and September. On the other hand, FEC taken at 1 or 2 months of age showed no, or weakly negative, correlations with later FEC. Lamb weight was weakly negatively correlated with some FEC and Peps, whereas there were some weak positive correlations between IgA and pepsinogen.

### Univariate analysis: worm length

There were significant relationships between a variety of variables and worm length (Table 3). Lambs with longer worms had higher FEC (FEC.4 and FEC.5), higher body weights (WT.4 and WT.5) but lower IgA activity (IgA.4 and IgA.5) and lower pepsinogen (Peps.). Pepsinogen had the strongest correlation with worm length (−0·34). The similar RMSEP showed that the variables had similar predictive abilities (Table 3).

### Univariate analysis: worm number

Table 4 shows that there were significant associations between the measurements on live lambs and worm number. FEC in October (FEC.6), IgA in August (IgA.4) and Peps were positively associated with worm number; the correlation of Peps was higher than FEC. Lamb weights (WT.2, WT.3, WT.4 and WT.5) were negatively correlated with worm number. There was a little difference in the predictive ability of each significant variable. However, FEC with a quadratic term in October (FEC.6+FEC.6^2^) produced slightly lower values of RMSLEP (Table 4, RMSLEP).

### Multivariate analysis: worm length

The predictabilities (the mean values of RMSEP) for all 6 models relating to worm length are given in Table 5. There was no difference between multiple regression and CPCR with the reduced set of variables (models MR1_Length, MR2_Length, CPCR1_Length and CPCR2_Length). The MR with the full set of variables (MR3_Length) produced the widest confidence interval for RMSEP. There was little difference in the distributions of RMSEP using CPCR with the full set of variables (CPCR3_Length) and the remaining 4 models.

**Table 5.** Predictability of models relating to worm length, measured using RMSEP, and predictability of models relating to worm number, measured using RMSLEP, for each model listed in Table 2

Model code	Variables	Predictability	95% confidence interval
MR1_Length	Combination of sex, pepsinogen, fecal egg count and IgA in October, weight and eosinophil in September that reduced RMSEP	0·013	(0·011, 0·015)
CPCR1_Length	Combination of Sex, pepsinogen, fecal egg count and IgA in October, weight and eosinophil in September that reduced RMSEP	0·013	(0·011, 0·015)
MR2_Length	Significant variables listed in Table 3	0·013	(0·011, 0·016)
CPCR2_Length	Significant variables listed in Table 3	0·013	(0·011, 0·015)
MR3_Length	All variables	0·017	(0·012, 0·411)
CPCR3_Length	All variables	0·014	(0·011, 0·018)
MR1_Number	Combination of sex, pepsinogen, fecal egg count and IgA in October, weight and eosinophil in September that reduced RMSLEP	0·760	(0·624, 0·905)
CPCR1_Number	Combination of Sex, pepsinogen, fecal egg count and IgA in October, weight and eosinophil in September that reduced RMSLEP	0·770	(0·625, 0·945)
MR2_Number	Significant variables listed in Table 4	0·897	(0·625, 0·922)
CPCR2_Number	Significant variables listed in Table 4	0·812	(0·630, 0·945)
MR3_Number	All variables	0·767	(0·683, 5·703)
CPCR3_Number	All variables	0·760	(0·663, 1·011)

The indices from models CPCR3_Length and MR1_Length were used to compare the full set of variables with just the final measurement of each variable. In each case, worm length was predicted as a weighted linear combination of the standardized indicator traits (Table 6).

**Table 6.** Variable weights for models CPCR3_Length, MR1_Length, CPCR3_Number and MR1_Number. The last row gives the correlations between each of the four resulting indices and the observed values. Variables with the largest weights are highlighted in bold

Variable	CPCR3_Length	MR1_Length	CPCR3_Number	MR1_Number
Constant	0·874	0·816	8·170	9·047
Sex	0·006		−0·110	−0·418
FEC.1 (FEC.1^2^)	0·007 (−0·001)		−0·050 (−0·001)	
FEC.2 (FEC.2^2^)	−0·003 (−0·004)		−0·011 (−0·001)	
FEC.3 (FEC.3^2^)	−0·005 (0·007)		−0·139 (0·100)	
FEC.4 (FEC.4^2^)	**0·030 (−0·021)**		−0·171 (0·113)	
FEC.5 (FEC.5^2^)	**0·034 (−0·030)**		−0·081 (−0·033)	
FEC.6 (FEC.6^2^)	**0·073 (−0**·**049)**	0·0001	**0·643 (−0·373)**	0·001
IgA.4	−0·013		0·066	
IgA.5	−0·009		−0·024	
IgA.6	0·019		−0·014	
WT1	0·008		0·030	
WT2	−0·003		0·066	
WT3	**−0·041**		0·097	
WT4	**0·052**		**−0·123**	
WT5	0·014	0·002	**−0·154**	−0·046
Peps	**−0·037**	−0·002	**0·244**	0·010
EOS1	−0·010		0·034	
EOS2	0·012		0·071	
EOS3	−0·005		−0·007	
EOS4	−0·002		−0·018	
EOS5	0·002		0·027	−0·002
IgA.4 × FEC.4	−0·002		0·033	
IgA.5 × FEC.5	−0·005		−0·003	
IgA.6 × FEC.6	**−0·039**		−0·073	
Correlation with observed values	0·55 (0·48, 0·60)	0·44 (0·37, 0·51)	0·51 (0·44, 0·57)	0·46 (0·39, 0·53)

**Fig. 2.**
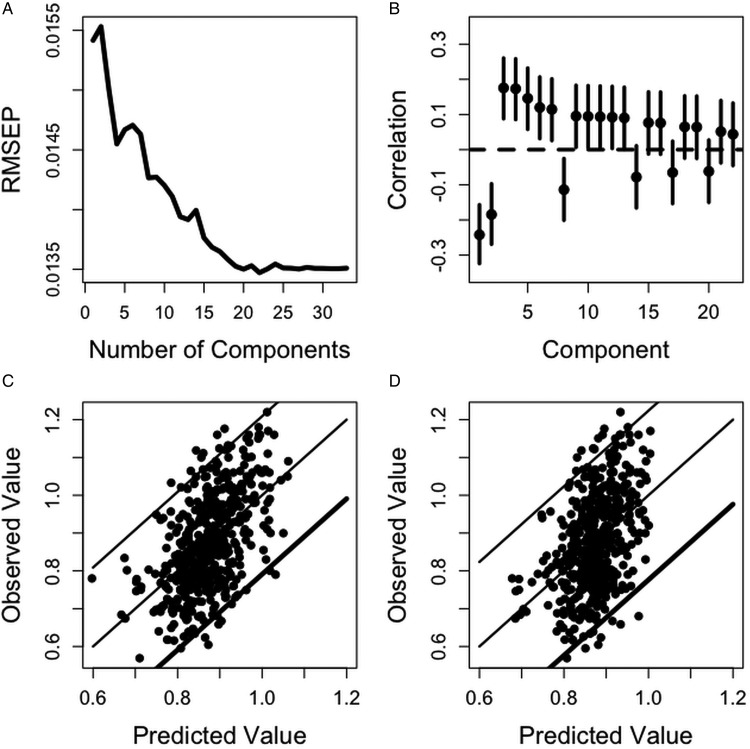
(A) RMSEP for worm length prediction using different numbers of components in CPC regression. This value is minimised using 22 components. (B) Correlations between the first 20 components and worm length (dots) and 95% confidence intervals (solid lines). (C) Predicted worm length using 20 components in CPC regression (CPCR3_Length) plotted against observed worm length with fitted line and prediction intervals (solid lines) and the line of equality (dotted line). (D) Predicted worm length using the minimal set of traits (MR1_Length) plotted against the observed worm length with fitted line and prediction bands (solid lines) and the line of equality (dotted line).

Using the full dataset (CPCR3_Length), we found that the model with all 22 principal components minimized the median RMSEP (Fig. 2A), however not all these components were significantly correlated with worm length. The correlation between the leading principal component and worm length was equal to −0·24 (Fig. 2B). The first rotated component is a weighted average of FEC measured between August and October. The second component had a correlation of −0·18 with worm length and the rotated component is a weighted average of the three IgA measurements and eosinophilia measured in September.

With all the 22 components, the index for worm length is heavily weighted by FEC from August, September and October, lamb weight in July and August, Peps and the interaction between IgA and FEC in October (Table 6, CPCR3_Length). The correlation between the predicted and observed worm length was 0·55 and significantly different from zero (*p*-value <0·0001, confidence interval 0·50, 0·62).

Using only the final measurements, we found little difference in comparing models MR1_Length and CPCR1_Length. Therefore, in model MR1_Length, we used cross-validation to determine the minimal set of traits that minimized the RMSEP. The set of variables included sex, FEC and pepsinogen measured in October and weight measured in September (Table 6). The correlation between the observed and predicted worm lengths using this index was 0·44 and significantly different from zero (*p*-value <0·0001, confidence interval 0·37, 0·51).

Prediction bands were computed over the range of observed worm lengths using models CPCR3_Length (Fig. 2C) and MR1_Length (Fig. 2D). In both plots, the solid middle line shows the best fitting line and the line of equality. The outer two lines give prediction bands. Most notably, the reduced set of traits (MR1_Length, Fig. 2D) does not predict that any worms will not be longer than 1 cm. The prediction bands using model CPCR3_ Length are narrower than those using model MR1_Length. For example, an index value of 1 cm had a predicted observed value between 0·79 and 1·20 cm using CPCR3_Length and a predicted observed value between 0·77 and 1·22 cm using MR1_Length.

### Multivariate analysis: worm number

We compared the predictive ability of the 6 possible models relating to worm number using RMSLEP (Table 5). We observed a similar pattern to worm length; the multiple regression with the full set of variables (MR3_Number) produced a wider range of RMSLEP values than the remaining 5 models.

The indices from models MR1_Number and CPCR3_Number were compared (Table 6). In each case, worm number is predicted as the exponential of a weighted linear combination of the standardized indicator traits (Table 6).

**Fig. 3.**
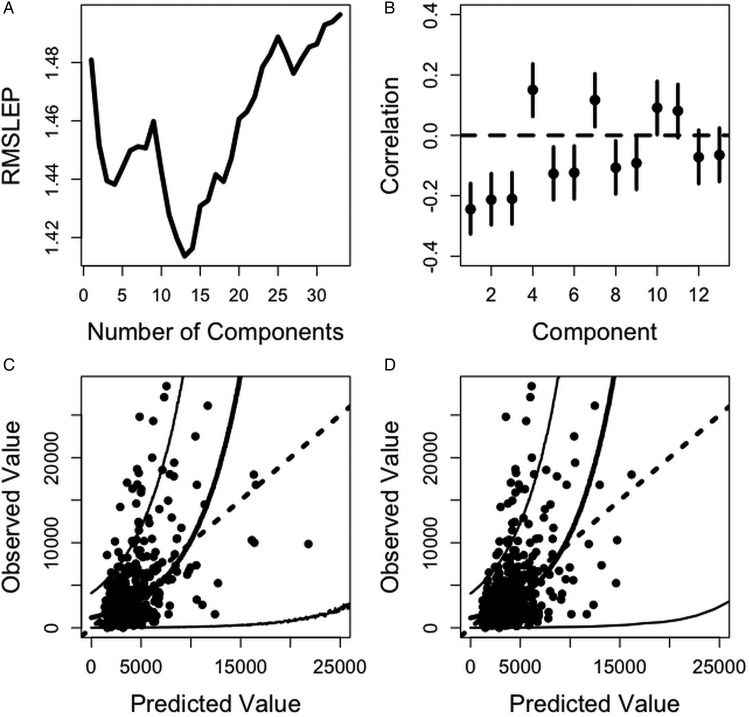
(A) RMSLEP for worm number prediction using a range of component numbers in CPC regression. This value is minimized using 13 components. (B) Correlations between the first 18 components and worm number (dots) and 95% confidence intervals (solid lines). (C) Predicted worm number values using 18 components in CPC regression (CPCR3_Number) plotted against observed worm number with fitted line and prediction intervals (solid lines) and the line of equality (dotted line). (D) Predicted worm number using the minimal set of traits (MR1_Number) plotted against observed worm number with fitted line and prediction intervals (solid lines) and the line of equality (dotted line).

In model CPCR3_Number, 13 principal components minimized the RMSELP (Fig. 3A). The correlation between worm number and the first component was −0·24 and the correlation with the second component was −0·21 (Fig. 3B). We found the first rotated component to be a weighted average between Peps and IgA in August and the interaction between FEC and IgA in August. The second component was a weighted average of FEC in July and August and lamb weight in August and September. The third rotated component was a weighted average of the five lamb weights. FEC in July, August and October, Peps in October and lamb weight in August and September are the most heavily weighted in the index (Table 6). The correlation between the predicted worm numbers (using CPCR3_Number) and the observed worm numbers was 0·51 and significantly different from zero (*p*-value <0·0001; confidence interval 0·53, 0·64).

Using cross-validation, the final index included FEC and pepsinogen measured in October and weight and eosinophil measured in September (Table 6). The correlation between the predicted worm numbers (using MR1_Number) and observed worm numbers was 0·46 and significantly different from zero (*p*-value <0·0001; confidence interval 0·39, 0·53).

Prediction bands were computed over the range of observed worm numbers using models CPCR3_Number (Fig. 4C) and MR1_Number (Fig. 4D). In both cases, these bands are wide and indicate that these predictive indices are not very informative about worm number. However, we found model CPCR3_Number to be slightly more accurate and precise in predicting larger worm numbers corresponding to the most heavily infected lambs. For example, an index value of 10 000 would predict a worm count between 106 and 35 294 with a point estimate of 10 273 using model CPC3_Number, whereas using model MR1_Number would predict an observed worm number between 106 and 38 633 with a point estimate of 11 057.

## DISCUSSION

We quantified the intensity of *T. circumcincta* infection in Scottish Blackface lambs using a range of indicator traits and devised linear combinations of these measurable traits that were highly correlated with the intensity of infection.

MR can be used for both inference and prediction. Raadsma *et al*. (2008) used MR to develop a predictive index for *Fasciola gigantica* in sheep and cattle. Although this method can produce near perfect results within the training data, it performs poorly when predicting observations out with the training set, a consequence of overfitting. We used correlation principal components regression to make linear combinations of the set of indicator traits, and then formed a predictive index for infection based on those linear combinations most correlated with infection. By assessing which traits were more heavily weighted in the leading few components, we were able to detect traits which are most useful in predicting infection. Predictive indices based on this method were better predictors and more correlated to worm number and length than any individual trait (Tables 3 and 4).

FECs measured between August and October were heavily weighted in the leading rotated component, most correlated with average worm length, whereas IgA and eosinophils were heavily weighted in the second rotated component. IgA activity has previously been shown to be associated with FEC and reduced adult female worm length (Strain *et al*. 2002). Worm length is highly correlated with fecundity (Stear *et al*. 1997) and IgA appears to be a major mechanism regulating the worm growth (Stear *et al*. 1999*b*). Univariately, our analysis is consistent with the previous work since we found plasma IgA to be negatively correlated with worm length. However, plasma IgA, FEC and pepsinogen were similar in their ability to predict worm length (Table 3). The resulting predictive index (CPCR3_Length) is mostly influenced by FEC, lamb weight and Peps and has a relatively strong correlation of 0·55 with worm length.

Using measurements for each trait taken in the last month (MR1_Length) failed to accurately predict lambs with an average worm length >1 cm (Fig. 2D), which corresponds to lambs least able to suppress worm development. The index derived, using all available data (CPCR3_Length) had a stronger correlation with worm length than any individual trait or the index derived using multiple traits taken in the last month of the study (MR1_Length, Table 6). Therefore, it is beneficial to monitor changes in FEC and weight over the final few months in order to best predict a lamb's ability to regulate worm length.

Predicting worm number presented the biggest challenge, partly due to the level of dispersion in these data. Using the full set of variables in a correlation principal components regression (CPCR3_Number) produced the most precise predictions; FEC and Peps in October and lamb weight in the final 2 months were heavily weighted in the predictive index (Table 6). The correlation between the predicted and observed worm numbers was 0·51, whereas using only the last measurement on each trait (MR1_Number, Table 6) produced a lower correlation of 0·46. The latter index also produced slightly wider prediction bands (Fig. 3C and D).

There was a clear density-dependent effect of worm number on worm length and lambs fell into one of the three categories. They either showed a large number of small worms, a small number of large worms or a small number of small worms. An increase in worm number may stimulate an immune response and a rise in concentration of pepsinogen in the plasma, which is also a characteristic of infection and is strongly associated with the decreased mean worm length (Table 3, Stear *et al.* 1999*a*). Since the pathogenic effects of *T. circumcincta* depend on both the length and the number of worms (Stear *et al*. 2003), creating an index to predict a combination of worm length and number, may better identify lambs most resistant to nematode infection.

The most informative indices are those that use information from a variety of tests that are measured on several occasions. The cost of multiple tests may prevent multivariable indices being used widely on many farms, although, they may have a role in breeding farms to help identify superior rams. However, their main role may be in experimental studies where it is desirable to monitor changes in the intensity of infection over time. Currently the information from multiple tests is interpreted based on subjective beliefs of the scientist. Indices provide a rigorous and useful way to combine information objectively. For instance, a multivariable index could be used as an indicator trait in a variety of procedures, for example searching for QTL, identifying resistant animals for breeding, or comparing the effect of different control procedures.

The indices in this paper have used information from Scottish blackface lambs naturally infected with predominantly *T. circumcincta.* Further research is required to determine indices for other breeds or nematode populations. However, the methods developed in this paper provide a template for constructing future indices.

## CONCLUSION

We have shown that combining measureable traits is more informative in predicting *T. circumcincta* resistance in Scottish Blackface lambs than using individual traits. Furthermore, this comprehensive dataset allowed us to assess the advantages of repeated measures in predicting resistance. Using the full dataset, we were able to more precisely and accurately predict worm length and number than only considering single measures of each trait taken at the end of the grazing season.
